# Non-linear relationships between inflammatory indices and erectile dysfunction in a group of young men living with HIV

**DOI:** 10.1186/s12610-026-00320-6

**Published:** 2026-06-24

**Authors:** Giorgio Tiecco, Matteo Riva, Federico Cesanelli, Luca Rossi, Cosimo Colangelo, Stefania Agata Reale, Federica Gottardi, Andrea Delbarba, Carlo Cappelli, Emanuele Focà, Francesco Castelli, Eugenia Quiros-Roldan

**Affiliations:** 1 Department of Clinical and Experimental Sciences, Unit of Infectious and Tropical Diseases, University of Brescia and ASST Spedali Civili di Brescia, Brescia, 25123 Italy; 2 Department of Medical and Surgical Specialties, Radiological Sciences and Public Health, University of Brescia, ASST Spedali Civili of Brescia, Brescia, Italy; 3 Department of Medicine, Unit of Endocrinology and Metabolism, ASST Spedali Civili Brescia, Brescia, Italy; 4 Department of Global Public Health, Karolinska Institutet, Stockholm, Sweden

**Keywords:** Erectile dysfunction, HIV, Young, Indices, Atherogenic indices, Atherosclerosis, NLR, Dysfonction érectile, VIH, Jeunesse, Indices, Indices athérogènes, Athérosclérose, RPN

## Abstract

**Background:**

Erectile dysfunction (ED) is a prevalent but underrecognized condition in young men living with HIV (yMLWH). Despite evidence linking inflammation and endothelial dysfunction to ED in the general population, little is known about these associations in yMLWH. This study aims to evaluate the relationship between novel inflammatory indices and ED in yMLWH.

**Results:**

This cross-sectional single-center study included yMLWH aged 18–50 years attending the HIV outpatient clinic at ASST Spedali Civili di Brescia, Italy, between January 2023 and January 2024. ED was assessed by self-report during routine clinical visits. Associations between inflammatory indices and ED were explored using generalized additive (GAM) and generalized linear models (GLM). Among 308 participants, 63 (20.5%) reported ED. Demographic characteristics and viro-immunological parameters were comparable between participants with and without ED; however, individuals with ED showed a higher prevalence of ≥ 3 comorbid conditions (12% vs. 7%), particularly cardiovascular disease (*p* = 0.018), dyslipidaemia (*p* < 0.001), and hepatic steatosis (*p* < 0.001).No significant differences were observed in inflammatory or atherogenic indices between participants with and without ED. Neither univariate nor multivariate models identified significant associations between indices and ED. Although GAMs revealed mildly nonlinear trends for atherogenic index of plasma (AIP), platelet-to-lymphocyte ratio (PLR), and platelet-to-neutrophil ratio (PNR), none reached statistical significance, though model fit was better than for linear models.

**Conclusions:**

In contrast to findings in the general population, no significant associations were observed between ED and inflammation-based indices in our cohort, potentially reflecting HIV-specific mechanisms or age-related differences in index expression. These results support the relevance of non-inflammatory contributors to ED in yMLWH and emphasize the need for multidisciplinary assessment.

**Supplementary Information:**

The online version contains supplementary material available at 10.1186/s12610-026-00320-6.

## Résumé

**Contexte:**

La dysfonction érectile (DE) est une affection répandue mais sous-estimée chez les jeunes hommes vivant avec le VIH. Malgré les preuves liant inflammation et dysfonctionnement endothélial à la DE dans la population générale, on ne sait que peu de choses sur ces associations chez les jeunes hommes vivant avec le VIH. Cette étude vise à évaluer la relation entre de nouveaux indices inflammatoires et la DE dans cette population.

**Résultats:**

Cette étude transversale monocentrique a inclus des jeunes hommes vivant avec le VIH, âgés de 18 à 50 ans, suivis en ambulatoire à la clinique ASST Spedali Civili di Brescia, en Italie, entre janvier 2023 et janvier 2024. La DE a été évaluée par auto-déclaration lors de visites cliniques de routine. Les associations entre indices inflammatoires et DE ont été explorées à l’aide de modèles additifs généralisés (MAG) et linéaires généralisés (MLG). Parmi les 308 participants, 63 (20,5%) ont déclaré une DE. Les caractéristiques démographiques et les paramètres viro-immunologiques étaient comparables entre les participants avec et sans DE ; cependant, les individus atteints de DE présentaient une prévalence plus élevée d’au moins 3 affections comorbides (12% versus 7%), en particulier les maladies cardiovasculaires (p = 0,018), la dyslipidémie (p < 0,001) et la stéatose hépatique (p < 0,001). Aucune différence significative n’a été observée dans les indices inflammatoires ou athérogènes entre les participants avec et sans DE. Ni les modèles univariés ni les modèles multivariés n’ont identifié d’associations significatives entre les indices et la DE. Bien que les MAG aient révélé des tendances légèrement non linéaires pour l’indice athérogène du plasma, pour le rapport plaquettes/lymphocytes et pour le rapport plaquettes/neutrophiles (RPN), aucun n’a atteint de signification statistique, bien que l’ajustement des modèles ait été meilleur que pour les modèles linéaires.

**Conclusions:**

Contrairement aux résultats de la population générale, aucune association significative n’a été observée entre la DE et les indices basés sur l’inflammation dans notre cohorte, ce qui reflète potentiellement des mécanismes spécifiques au VIH ou des différences liées à l’âge dans l’expression de ces indices. Ces résultats confirment la pertinence des contributeurs non inflammatoires à la DE chez les jeunes hommes vivant avec le VIH, et soulignent la nécessité d’une évaluation multidisciplinaire.

## Introduction

Erectile dysfunction (ED), defined as the persistent inability to attain or maintain an erection, is a prevalent yet underrecognized condition among aging males [1]. A significant gap exists between self-reported ED and clinical diagnosis, as many affected individuals do not seek medical care [1]. While psychological and interpersonal factors are particularly relevant in younger men [2], numerous organic factors (including obesity, type 2 diabetes, smoking, alcohol use, insomnia, depression, and hypertension) have been consistently associated with ED, even in recently published Mendelian randomization studies [3]. These conditions often converge on endothelial dysfunction and atherosclerosis.

In this context, several novel and easily obtainable inflammatory and atherogenic indices [4], such as neutrophil-to-lymphocyte ratio (NLR), platelet-to-lymphocyte ratio (PLR), lymphocyte-to-monocyte ratio (LMR), neutrophil-to-lymphocyte-platelet ratio (NLPR), and atherogenic index of plasma (AIP), have been proposed as potential predictors of ED in the general population [4–6]. Among them, NLR has shown a particularly robust association with ED in large cross-sectional studies, although no standardized cut-off exists and reported thresholds vary across studies [7–9]. Furthermore, in combination with AIP, NLR improves the predictive accuracy for cardiovascular diseases, highlighting its potential utility in risk stratification [10].

Several studies report that the prevalence of ED among people living with HIV (PLWH) ranges from 13% to 86%, consistently exceeding rates observed in age-matched HIV-negative populations [11, 12]. While antiretroviral therapy (ART) derived adverse effects have historically been implicated, currently the pathogenesis of ED in PLWH appears to be more closely linked to chronic immune activation and systemic inflammation [13, 14]. Recent observational studies using large-scale genetic data have further supported a strong association between circulating immune cell profiles and ED, reinforcing the potential immunological basis of ED [15]. Similarly to the general population, inflammation-based ratios have gained recognition as valuable indices for assessing immune dysregulation in PLWH [16]. Despite this growing interest, no study to date has comprehensively evaluated these novel indices in PLWH with ED.

This cross-sectional, single-center study investigates the association between inflammatory indices with ED in a cohort of young men living with HIV (yMLWH) routinely followed at an outpatient HIV clinic of a tertiary care hospital in Northern Italy.

## Participants and methods

### Study design and participants

This cross-sectional, single-center study was conducted in a cohort of yMLWH, routinely followed at the outpatient HIV clinic of ASST Spedali Civili di Brescia, Italy. All individuals aged 18 to 50 were systematically screened for ED symptoms during routine follow-up visits, in accordance with the most recent European AIDS Clinical Society (EACS) guidelines [17]. Inclusion criteria were confirmed HIV infection and age between 18 and 50 years, with the upper limit set to minimize age-related confounding factors associated with ED. Exclusion criteria included current psychiatric disorders requiring treatment, previous use of antidepressant or antipsychotic medications, current or previous chemotherapy within the previous 2 years, current or occasional use of phosphodiesterase type 5 inhibitors, current use of gonadotropin-releasing hormone analogues, finasteride, cyproterone, or flutamide, and current use of anticonvulsant medications. During routine follow-up visits, individuals declining participation or subsequently identified as having an active psychiatric disorder requiring treatment were further excluded. Participants were stratified into two groups: those reporting symptoms of ED and those without.

### Definitions

ED was defined as any self-reported persistent difficulty in attaining or maintaining an erection, regardless of severity. HIV viral suppression was defined as plasma HIV-RNA < 50 copies/mL at the time of the visit. Derived inflammatory and atherogenic indices were calculated as follows. NLR was defined as the neutrophil count divided by the lymphocyte count [18], while the PLR was calculated as the platelet count divided by the lymphocyte count [19]. The PNR was defined as the platelet count divided by the neutrophil count, and the LMR as the lymphocyte count divided by the monocyte count [20]. The NLPR was calculated as the neutrophil count multiplied by 100 and divided by the product of lymphocyte and platelet counts [21]. Finally, the AIP was defined as the base-10 logarithm of the ratio between fasting triglyceride and high-density lipoprotein cholesterol (HDL) [22].

### Data collection

Viro-immunological data were collected for all enrolled participants, including duration of HIV infection, HIV viral load and immunological profile at the time of the visit. Clinical data on the following comorbidities were also collected: cardiovascular disease, diabetes, renal dysfunction, dyslipidaemia, hepatic steatosis, osteoporosis, HCV infection (treated or ongoing), and HBV status (occult HBV infection or chronic HBV infection/hepatitis). Current chronic alcohol consumption, and current smoking were also collected. Definitions were based on clinical evidence and/or active treatment. The most recent laboratory results were also recorded, comprising white blood cell count (WBC), platelet count, neutrophils, monocytes, lymphocytes, total cholesterol, HDL, calculated low-density lipoprotein (LDL), and triglycerides. All variables were stratified according to the presence or absence of ED.

### Ethics

The study protocol was approved by the Comitato Etico Territoriale Lombardia 6, Fondazione I.R.C.C.S. Policlinico San Matteo, 27,100 Pavia, Italy (protocol number 0018047/25) and conducted in accordance with the ethical standards of the Helsinki Declaration (1975, revised in 2013). Written informed consent has been obtained from each subject.

### Statistical analysis

For the descriptive analysis, we divided the sample into two groups according to the presence or absence of ED. Categorical variables were summarized as frequencies and percentages, while continuous variables were reported as means with standard deviations. Continuous variables were compared using the Welch’s t-test (for parametric variables) or the Mann-Whitney U test (for non-parametric variables) depending on their distribution; normality was assessed using the Shapiro-Wilk test. Categorical variables were compared using the chi-squared test. After an exploratory data analysis, to investigate potential linear associations between each inflammatory and atherogenic index (NLR, PLR, NLPR, LMR, AIP, PNR and CD4/CD8 ratio) and ED, we employed a generalized additive model (GAM) with a binomial distribution and logit link. GAMs were fitted without additional covariate adjustment. Potential confounders were selected based on clinical relevance and statistical considerations. In order to avoid overfitting and given the limited number of outcome events, clinically relevant variables but not statistically significant were not included in the analysis. When CD4/CD8 ratio was considered in the analyses, CD4 + and CD8 + counts were excluded to avoid collinearity. To investigate more thoroughly the linear effects of each inflammatory index, we computed and plotted marginal effects of each variable related to predicted probabilities from the GAM model. Predicted probabilities refer to the estimated probability of ED derived from the fitted GAM models. A linear model was also evaluated; each index was analysed individually using both univariate GAMs and generalized linear models (GLMs). For each pair of models, Akaike Information Criterion (AIC) values were compared to evaluate model fit. Odds ratios with 95% confidence intervals were calculated for the GLM models. Comorbidity burden was assessed using an unweighted cumulative score obtained by summing the comorbid conditions recorded for each participant and was included in multivariable models as a covariate. A multivariable logistic regression model was also fitted to adjust for potential confounding factors, including age, total CD4 + and CD8 + T-cell counts, and comorbidity burden. No formal sample size calculation was performed, as this study was designed as an exploratory pilot study. The sample size was therefore based on the number of eligible participants attending the clinic during the study period. All statistical tests were two-sided, and a p-value < 0.05 was considered statistically significant. All statistical analyses were performed using R statistical software [23].

## Results

A total of 358 participants were screened for eligibility. During routine follow-up visits, 24 (6.7%) were further excluded due to psychiatric disorders requiring treatment and 26 (7.3%) declined participation. Overall, 308 participants were included in the analysis (Supplementary Fig. 1), of whom 63 (20.5%) reported ED with a mean International Index of Erectile Function-5 (IIEF-5) score of 13.6 (± 5.9). Demographic features and viro-immunological parameters were comparable between participants with and without ED. However, patients with ED had a significantly higher comorbidity burden compared to those without ED. Specifically, 12% of patients with ED reported at least three comorbidities, compared to 7% of patients without ED. The most frequent comorbidities were cardiovascular diseases (25.4% vs. 13.1%, *p* = 0.018), dyslipidaemia (57.1% vs. 32.2%, *p* < 0.001), and hepatic steatosis (35.2% vs. 12.4%, *p* < 0.001). No statistically significant differences were observed in inflammatory or atherogenic indices between the two groups. The descriptive analysis of the study population is extensively reported in Table 1.

**Table 1:** Baseline characteristics of the study population stratified by erectile dysfunction status

	Reporting ED	Not-reporting ED	*p*-value
	Sample, *n* (%)	63 (100)	245 (100)
Age, mean (± SD)	44.16 (4.69)	42.93 (5.79)	0.258
Comorbidities			
Cardiovascular, *n* (%)	16 (25.4)	31 (13.1)	0.029
Dyslipidemia, *n* (%)	36 (57.1)	76 (32.2)	< 0.001
Hepatic steatosis, *n* (%)	19 (35.2)	27 (12.4)	< 0.001
Diabetes, *n* (%)	7 (11.11)	7 (2.97)	0.006
Renal dysfunction, *n* (%)	7 (11.11)	6 (2.54)	0.003
Osteoporosis, *n *(%)	1 (1.59)	10 (4.24)	0.323
HCV chronic infection (treated or ongoing), *n* (%)	11 (17.46)	31 (13.14)	0.382
HBV status			0.168
Occult HBV infection	6 (9.52)	41 (17.37)	
chronic HBV infection/hepatitis	1 (1.59)	3 (1.27)	
Comorbidity burden, median (range)	3 (0–7)	2 (0–6)	< 0.001
Lifestyle habits			
Smoking, *n* (%)	27 (44.26)	107 (47.56)	0.649
Drinking, *n* (%)	37 (60.66)	84 (38)	0.002
Viro-immunological markers			
Years of infection, *n* (%)	12.7 (6.9)	13.3 (6.8)	0.432
HIV-RNA undetectable, *n* (%)	59 (95.2)	233 (98.7)	0.075
CD4 cells, mean (± SD)	860 (408)	922 (366)	0.139
CD4%, mean (± SD)	33.0 (10.2)	34.8 (9.1)	0.193
CD8 cells, mean (± SD)	1019 (475)	984 (416)	0.821
CD8%, mean (± SD)	39.4 (10.1)	36.9 (9.7)	0.066
CD4/CD8 ratio, mean (± SD)	0.9 (0.5)	1.1 (0.5)	0.092
Laboratory assays			
WBC, mean (± SD)	6.8 (1.9)	6.7 (1.8)	0.680
PLT, mean (± SD)	239,918 (67,493)	243,639 (62,192)	0.268
Neutrophil count, mean (± SD)	3.7 (1.5)	3.5 (1.5)	0.336
Neutrophil%, mean (± SD)	52.6 (12.8)	52.0 (10.5)	0.389
Monocyte count, mean (± SD)	586 (177)	628 (709)	0.788
Monocyte%, mean (± SD)	8.8 (1.8)	9.8 (14.1)	0.968
Lymphocytes count, mean (± SD)	2.3 (0.9)	2.4 (0.7)	0.279
Lymphocytes%, mean (± SD)	35.3 (10.6)	35.6 (9.44)	0.810
Cholesterol, mean (± SD)	185 (39.9)	184 (35.1)	0.848
HDL, mean (± SD)	46.5 (12.5)	47.4 (13.1)	0.348
LDL, mean (± SD)	117 (31.4)	118 (30.5)	0.857
TG, mean (± SD)	140 (95.7)	118 (71.2)	0.114
Inflammatory and atherogenic indices			
NLR, mean (± SD)	1.9 (1.1)	1.6 (0.9)	0.165
PLR, mean (± SD)	117.64 (53.11)	112.87 (48.75)	0.489
AIP, mean (± SD)	0.40 (0.22)	0.35 (0.22)	0.256
LMR, mean (± SD)	4.1 (1.6)	4.3 (1.5)	0.414
PNR, mean (± SD)	74.9 (35.1)	79.7 (43.6)	0.245
NLPR, mean (± SD)	0.82 (0.55)	0.70 (0.42)	0.119
Antiretroviral therapy			
Dual therapy, *n* (%)	132 (55.9%)	28 (45.2%)	0.171
INI based regimen, *n* (%)	213 (90.3%)	53 (85.5%)	0.396

Continuous variables were compared using the Welch’s t-test or the Mann-Whitney U test depending on their distribution, categorical variables were compared using the chi-squared test. Percentages are calculated considering missing data

*Abbreviations*  *ED* erectile dysfunction, *WBC* white blood count, *NLR* neutrophile-to-lymphocyte ratio, *PLR* platelet-to-lymphocyte ratio, *LMR* lymphocyte-to-monocyte ratio, *NLPR* neutrophil-to-lymphocyte-platelet ratio, *AIP *atherogenic index of plasma

Univariate logistic regression analyses did not identify any significant associations between ED and the indices examined (Table 2). Similarly, multivariate logistic regression models adjusting for age, comorbidity burden, and CD4/CD8 counts did not reveal any predictors of ED (Table 3).

**Table 2:** Univariate logistic regression models (GLM) assessing the association between inflammatory indices and erectile dysfunction

Variable	OR (95% CI)	*p*.value
NLR	1.22 (0.56–2.68)	0.622
PLR	1.00 (1.00–1.00)	0.974
NLPR	1.86 (0.37–9.50)	0.454
LMR	0.89 (0.59–1.33)	0.567
PNR	1.00 (0.98–1.01)	0.587
AIP	2.84 (0.47–17.02)	0.254
CD4/CD8	0.90 (0.41–1,97)	0.793

Erectile dysfunction as dependent variable

*Abbreviations*  *NLR* neutrophile-to-lymphocyte ratio, *PLR* platelet-to-lymphocyte ratio, *LMR* lymphocyte-to-monocyte ratio, *NLPR* neutrophil-to-lymphocyte-platelet ratio, *PNR* platelet-to-neutrophil ratio, *AIP* atherogenic index of plasma

**Table 3:** Multivariate logistic regression models (GLM) assessing the association between inflammatory indices and erectile dysfunction (adjusted analysis)

Variable	OR (95% CI)	*p*-value
NLR	0.74 (0.27–2.02)	0.552
PLR	1 (0.98–1.01)	0.499
NLPR	0.87 (0.11–6.97)	0.899
LMR	1.01 (0.62–1.65)	0.964
PNR	1 (0.98–1.02)	0.977
AIP	3.2 (0.39–26.04)	0.277
CD4/CD8	1.01 (0.43–2.43)	0.955

Erectile dysfunction as dependent variable. Covariates used: age (years), comorbidity burden, CD4 and CD8 counts (not used for CD4/CD8 ratio)

*Abbreviations*  *NLR* neutrophile-to-lymphocyte ratio, *PLR* platelet-to-lymphocyte ratio, *LMR* lymphocyte-to-monocyte ratio, *NLPR* neutrophil-to-lymphocyte-platelet ratio, *PNR* platelet-to-neutrophil ratio, *AIP *atherogenic index of plasma

To explore potential non-linear relationships, GAMs were fitted for each index. Given the absence of significant associations in the univariate models and to avoid overfitting, no additional covariates were included in the GAMs. None of the indices reached statistical significance in the GAM analyses (Table 4).

**Table 4:** Results from univariate generalized additive models (GAMs) evaluating non-linear associations between inflammatory indices and erectile dysfunction

Variable	*p*-value
NLR	0.515
PLR	0.383
NLPR	0.411
LMR	0.486
AIP	0.254
PNR	0.587
CD4/CD8	0.794

Erectile dysfunction as dependent variable. p-values derived from smooth terms in GAM models

*Abbreviations*  *NLR* neutrophile-to-lymphocyte ratio, *PLR* platelet-to-lymphocyte ratio, *LMR* lymphocyte-to-monocyte ratio, *NLPR* neutrophil-to-lymphocyte-platelet ratio, *PNR* platelet-to-neutrophil ratio *AIP* atherogenic index of plasma

Despite the lack of significant individual effects, the overall GAM model demonstrated a better fit compared to the corresponding GLM, as evidenced by a lower AIC (168.9 vs. 180.2) and a higher percentage of deviance explained (14.4% vs. 7.0%) (Table 5).

**Table 5:** Comparison of model performance between generalized additive models (GAMs) and generalized linear models (GLMs)

	GAM model	GLM model
AIC	168.8573	180.1855
Deviance explained	14.43%	7.00%

*Abbreviations*  *GAM* general additive model, *GLM* general linear model, *AIC* Aikaike’s information criteria, *NLR* neutrophile-to-lymphocyte ratio, *PLR* platelet-to-lymphocyte ratio, *LMR* lymphocyte-to-monocyte ratio, *NLPR* neutrophil-to-lymphocyte-platelet ratio, *AIP* atherogenic index of plasma

**Fig. 1:**
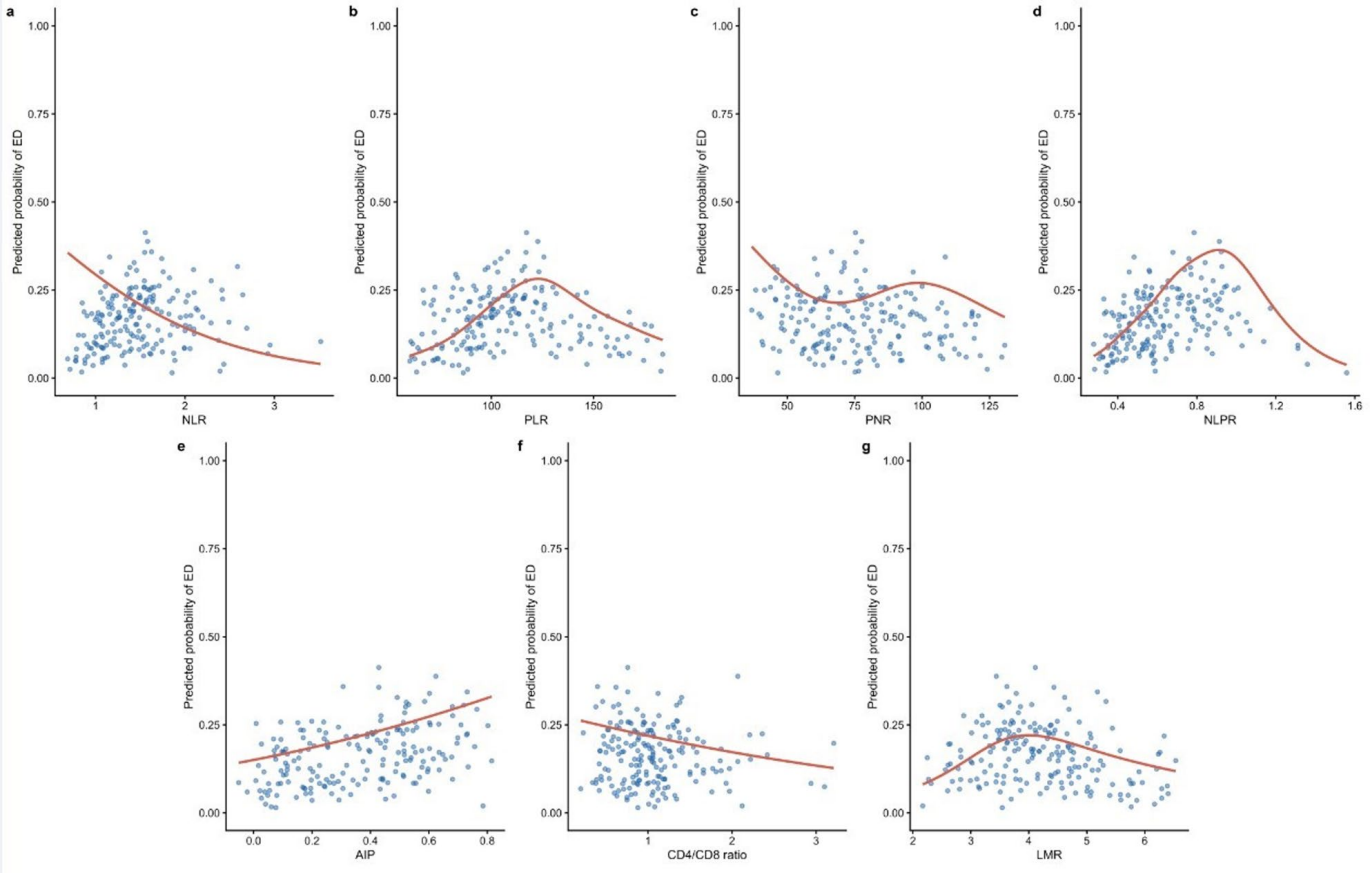
Marginal effects plots from a multivariable generalized additive model (GAM) showing the association between each inflammatory index and the predicted probability of erectile dysfunction (ED). Each panel represents the smoothed effect of one index while all others are held constant at their median values. Blue dots represent individual observations; the red line indicates the smoothed spline function estimated by the GAM. The y-axis represents the predicted probability of ED; the x-axis shows the value of each index. Statistical significance of each smooth term was evaluated using the approximate F-test (mgcv package, R). **a** Predicted probability of ED as a function of NLR. **b** Predicted probability of ED as a function of PLR. **c** Predicted probability of ED as a function of PNR. **d** Predicted probability of ED as a function of NLPR. **e** Predicted probability of ED as a function of AIP. **f** Predicted probability of ED as a function of the CD4/CD8 ratio. **g** Predicted probability of ED as a function of LMR. Abbreviations: ED, erectile dysfunction; GAM, generalized additive model; NLR, neutrophil-to-lymphocyte ratio; PLR, platelet-to-lymphocyte ratio; PNR, platelet-to-neutrophil ratio; NLPR, neutrophil-to-lymphocyte-platelet ratio; AIP, atherogenic index of plasma; LMR, lymphocyte-to-monocyte ratio

Marginal effect plots (Fig. 1), computed by varying one index at a time while holding the others at their median values, did not reveal any consistent or clinically meaningful trends. Nonetheless, mild non-linear patterns were noted for AIP, PLR, and PNR.

## Discussion

Our cross-sectional study adds to the growing body of evidence on the burden of ED in PLWH, showing a prevalence of 20.5% within a large cohort of individuals aged under 50 years. No significant associations were observed between ED and inflammatory or atherogenic indices in either univariate or multivariate analyses.

In our cohort, the prevalence of ED among young men living with HIV was 20.5%, lower than rates reported in recent studies from Italy (59.7%) and other countries (37.5%) [24, 25]. Although definitive data in this age group are lacking, our findings are comparable to rates from population-based self-reported surveys in the United States [26]. Given that ED was assessed by self-report, the findings may also reflect the well-documented reluctance of patients to discuss sexual health [27].

Contrary to findings from general population studies, no significant associations were observed between ED and inflammatory or atherogenic indices in our cohort [6, 7]. Although HIV-related mechanisms may contribute to differences in ED pathophysiology, our study design does not allow direct inferences regarding the role of HIV-specific factors, particularly in the absence of an HIV-negative comparison group. A recent machine learning analysis in a comparable cohort of PLWH and HIV-negative individuals under 60 years identified HIV status as an independent predictor of clustering based on multiple penile Doppler parameters, irrespective of age [13], potentially reflecting the chronic inflammation, immune activation, and immunosenescence characteristic of PLWH [28–30]. The CD4/CD8 ratio, a recognized index of immunosenescence and inflammation [31], and a prognostic indicator for various conditions, including cardiovascular disease [32], and cancer [33, 34], also failed to be associated with ED in our study. This lack of association may reflect age-specific effects, as the CD4/CD8 ratio has been linked to adverse outcomes primarily in older populations, as shown in the Swedish OCTO and NONA longitudinal studies [35, 36]. In the general population, recently published correlation analysis has demonstrated a significant negative association between AIP and IIEF-5 scores, with multivariate analysis identifying AIP as an independent predictor of ED [37]. In our cohort, however, no statistical significance was observed for AIP or NLPR likely reflecting the younger age and absence of obesity compared with the population studied by Sambel M, et al. [37]. Nevertheless, trends in AIP and NLPR in both GAM and GLM may suggest an association with ED and a role for endothelial dysfunction, with other associations possibly masked by the relatively young age of the cohort and its limited size. Moreover, the superior fit of GAMs over GLMs supports the idea that ED–index relationships may be non-linear, as suggested in another study on inflammatory indices [7].

Our findings suggest that ED in young men living with well-controlled HIV may be also driven by non-inflammatory mechanisms, underscoring the importance of considering psychological aspects, sleep disturbance, and other contributing factors when assessing sexual health in this population [24, 38]. The observed burden of comorbidities further indicates a need for a comprehensive evaluation, incorporating referral to andrology specialists and the use of appropriate instrumental diagnostic methods according to the international guidelines [39]. In our cohort, the high prevalence of dyslipidaemia (57%) combined with guideline-driven statin use in PLWH aged > 40 years [17, 40], may have attenuated detectable inflammatory indices in our cohort. This effect may reflect the pleiotropic properties of statins, which extend beyond lipid lowering to include antithrombotic, immunomodulatory, and anti-inflammatory actions [41]. Evidence on the impact of statins on inflammatory indices remains inconsistent and is largely derived from the general population. Some studies report significant short-term reductions in NLR and PLR following statin initiation, including findings from retrospective cohorts [41] and a randomized controlled trial [42]. In contrast, other studies have demonstrated no significant changes after longer treatment durations [43].

### Limitations of the study

Our findings should be interpreted in light of some limitations. First, the cross-sectional, single-center design precludes causal inference and may limit generalizability. Second, the relatively small number of ED cases may have reduced statistical power and precluded the inclusion of additional covariates in the multivariable model. Third, ED was assessed by self-report rather than by validated instruments such as the IIEF or IIEF-5, potentially introducing misclassification bias. In addition, no data were collected on hormonal status, psychological or sexual health factors, or concomitant medications. Although key covariates were included in the models, residual confounding cannot be excluded. Lastly, as social and relationship factors are debated factors influencing ED among PLWH [44], no data were collected regarding partner characteristics, number of sexual partners, or marital/relationship status. However, as previously reported in studies conducted in the same clinical setting [14], over 50% of yMLWH did not report an exclusive male-to-male sexual activity.

## Conclusions

Our study is the first to investigate the relationship between novel inflammatory and atherogenic indices and ED in yMLWH, finding no significant associations. These results highlight the potential role of non-inflammatory mechanisms—such as psychological or functional factors—in the pathogenesis of ED in this population. A multidisciplinary approach, including andrological evaluation, is warranted to ensure comprehensive care. 

## Supplementary Information

Supplementary Material 1.

## Data Availability

The data that support the findings of this study are available on request from the corresponding author. The data are not publicly available due to privacy or ethical restrictions.
