# Supplementary figures and images for: Non-linear relationships between inflammatory indices and erectile dysfunction in a group of young men living with HIV
Source: Basic Clin Androl. 2026 Jun 24;36:18. doi: 10.1186/s12610-026-00320-6 (PMC13292434; doi:10.1186/s12610-026-00320-6)

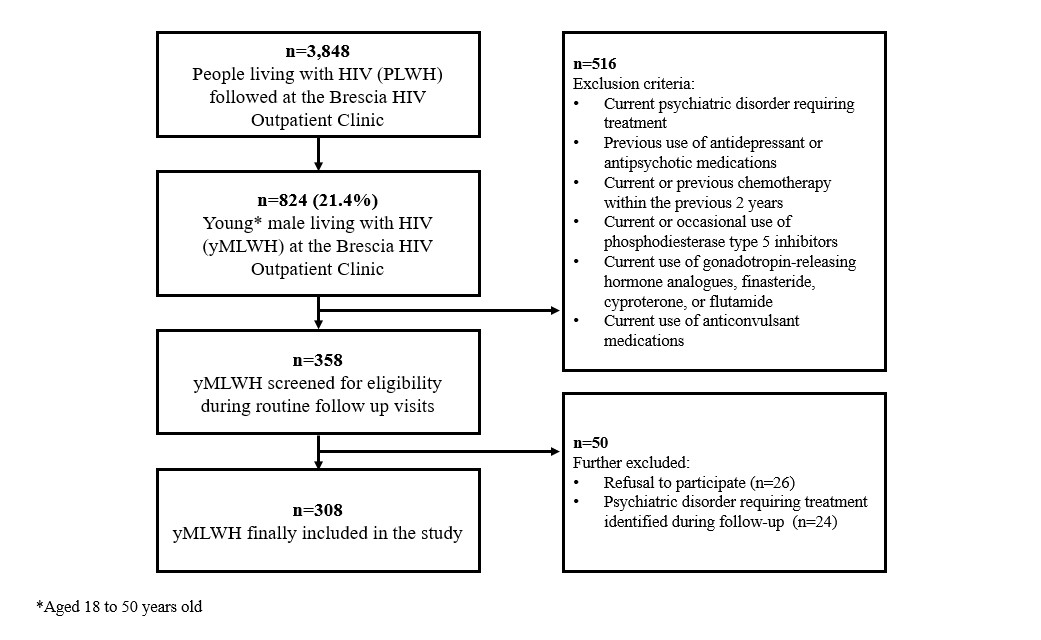

Supplement: Supplementary file 1 — Supplementary Material 1. [file 12610_2026_320_MOESM1_ESM.jpg]
